# Cross-cultural adaptation and validity of the Spanish fear-avoidance components scale and clinical implications in primary care

**DOI:** 10.1186/s12875-020-01116-x

**Published:** 2020-02-27

**Authors:** Antonio I. Cuesta-Vargas, Randy Neblett, Robert J. Gatchel, Cristina Roldán-Jiménez

**Affiliations:** 1 Department of Physiotherapy of the Faculty of Health Science at the University of Malaga, Cátedra de Fisioterapia, Universidad de Málaga, Andalucía Tech, Av/ Arquitecto Peñalosa, 3 (Teatinos Campus Expansión), Malaga, 29071 Spain; 2 School of Clinical Science, Faculty of Health at the Queensland University of Technology, Brisbane, Australia; 3 Instituto Investigación de Biomédica de Málaga (IBIMA), Málaga, Spain; 4 PRIDE Research Foundation, Dallas, TX USA; 5 Department of Psychology, Center of Excellence for the Study of Health & Chronic Illnesses, College of Science, The University of Texas at Arlington, Arlington, TX USA

**Keywords:** Fear-avoidance, The fear-avoidance components scale, FACS, Chronic musculoskeletal pain disorders, patient health questionnaire, decision making

## Abstract

**Background:**

Pain-related fear-avoidance (FA) is a common problem affecting many patients with painful medical conditions. As there is great interest in the clinical importance of the relationship between FA and disability, several questionnaires have been developed to measure FA. The Fear-Avoidance Components Scale (FACS) is a recently developed patient-reported instrument that addresses critical issues not previously considered in previous FA-related questionnaires. The original English version of the FACS demonstrated good reliability, internal consistency, and construct, criterion, and predictive validity. Two factors were determined: General Fear Avoidance and Types of Activities That are Avoided. The aim of this study was to to translate the FACS into European-style Spanish (FACS-Sp), and validate its psychometric properties.

**Methods:**

This two-stage psychometric study included 330 subjects with various chronic musculoskeletal pain disorders. An initial translation and cross-cultural adaptation of the FACS, from English to Spanish, was performed. Then, critical psychometric properties were analysed, including internal consistency by Cronbach’s α coefficients, structural validity from the Maximum Likelihood Extraction (MLE), and convergent validity by Pearson correlation with the Central Sensitization Inventory (CSI).

**Results:**

This study reports for the first time the psychometric properties of the Spanish version of the FACS. Total scores ranged from 0 to 88 points, with a mean of 30.49 (±17.18). The FACS-Sp showed a high internal consistency for factor 1 (α = 0.902) and factor 2 (α = 0.88). Factor structure was two-dimensional and supported structural validity, accounting for 48.75% of the total variance. Convergent validity analysis found a significant Pearson correlation r = 0.414.

**Conclusion:**

This study reports for the first time the psychometric properties of the Spanish version of the FACS-Sp. Psychometric properties supported the validation of FACS-Sp and ensured the conceptual equivalence with the original English version. In primary care and chronic pain rehabilitation, FA assessment is crucial for clinical decision-making and treatment guidance. The FACS-Sp offers a new measure of FA in Spanish speaking populations. Future research on the FACS-Sp should evaluate test-retest reliability, treatment responsiveness and psychometric comparisons with other translated versions.

## Background

Primary care has traditionally functioned within a biomedical model, which only considers the physical components of illness, and has tended to disregard psychosocial components [1]. One of the most influential models to explain psychological factors of pain is the fear-avoidance model [2]. Pain-related fear-avoidance (FA) is a common problem affecting patients with painful medical conditions. The relationship between pain and fear was first introduced in 1983 by Lethem et al. [2]. According to this FA model, after an injury, patients can respond to fear by confrontation or avoidance. Fear of pain and avoidance of activities can result in desynchronization of the actual sensory component of pain. An updated FA model that incorporated cognitive behavioral components was introduced in 1995 by Vlaeyen et al. [3]. Since then, the FA model has been corroborated and refined, explaining that if an experienced pain (with or without associated injury) is understood as a threat, and the patient begins to catastrophize, then pain-related fear can evolve, leading to avoidance of activities, hypervigilance, depression, physical disuse, deconditioning, and disability [4–6].

As there is great interest in the clinical importance of the relationship between pain-related FA and disability, several questionnaires have been developed to measure FA [7]. Patient-reported outcome measures (PROMS) [8, 9] are used to assess a patient’s symptoms and/or functional status at a specific time. Although PROMS data are subjective, they can help health care providers understand the patient’s subjective experiences, how a condition or disease influences a patient’s capabilities, and detect changes due to an intervention [10]. In the case of FA, several notable questionnaires have been published: the Tampa Scale for Kinesiophobia (TSK) [11], Pain Anxiety Symptoms Scale (PASS) [12], Pain Catastrophizing Scale (PCS) [13], and Fear-Avoidance Beliefs Questionnaire (FABQ) [14] among them. However, their construct validity has been criticized and very little support has been provided for treatment responsiveness [2]. Until recently, only the PASS and the TSK have cut-off scores available for clinical interpretation [7, 15]. In addition, their original versions were developed before the current FA model was fully developed, so none of them assesses all cognitive, emotional, and behavioral components of the model.

Recently, the Fear-Avoidance Components Scale (FACS) was developed, which incorporates important components of previous FA-related measures, and includes components of the FA model not previously considered in the earlier-developed questionnaires, within a framework of the most current FA model of Vlaeyen [6, 16]. The FACS has demonstrated acceptable test/retest reliability (*r* = .90–.94) and internal consistency (Cronbach *α* = .92). The original English version, and other translated versions of the FACS, are available at https://www.pridedallas.com/questionnaires/.

It is estimated that 14% of Europeans speak Spanish, which is about 50 million people [17]. Spanish versions of the TSK [18], PCS [19] and FABQ [20] are currently available, and there has recently been great deal of interest in using these instruments to assess components of pain-related FA among Spanish-speaking populations with chronic pain conditions, such as chronic migraine, temporomandibular disorders [21], knee and hip osteoarthritis [22] and chronic pelvic pain [23]. However, there is no published Spanish version of FACS. Hence, the goal of the present study was to translate the FACS into European-style Spanish (FACS-Sp), and validate its psychometric properties for its clinical use with native Spanish-speakers in Spain.

## Methods

### Design

This cross-sectional study was conducted in two stages. First, an initial translation and cross-cultural adaptation of the FACS, from English to Spanish, was performed. Secondly, for evaluation of the FACS-Sp’s critical psychometric properties, patient volunteers from a physical therapy outpatient clinic were used.

Psychometric properties were assessed according to the “COnsensus-based Standards for the Selection of health status Measurement INstruments” COSMIN guidelines [24]. Reliability, internal consistency, construct validity (cross-cultural and structural validity), and construct validity, in terms of convergent validity, were evaluated.

### Cross-cultural translation process

**Fig. 1:**
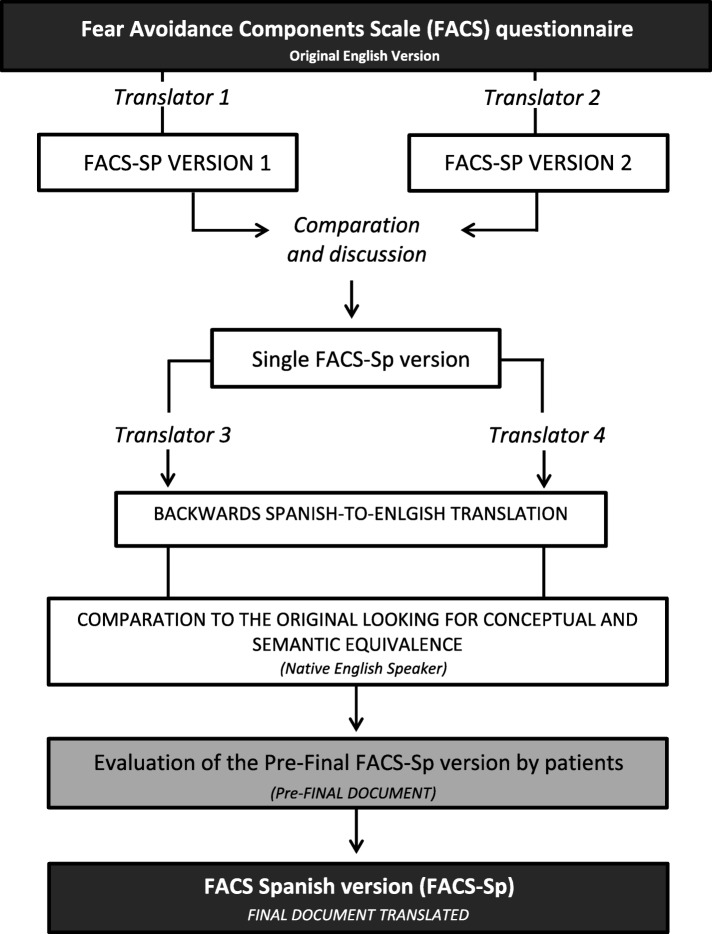
Flowchart of the development process FACS-Sp from the original version

An English–to-Spanish translation was carried out to ensure conceptual equivalence of all of the test items, while maintaining appropriate Spanish cultural linguistic qualities. For this purpose, a direct- and reverse-translation methodology was utilized, with the help of a specialist in the field, as recommended in the literature [25]. For this purpose, two independent English-to-Spanish translations were made by two separate translators (CRJ and DPC authors). This process produced two Spanish versions of the FACS-Sp. After discussion among the two participants, a single FACS-Sp version was reached. Then, a backward Spanish-to-English translation was agreed-upon by two blinded and independent professional Spanish translators, who were not familiar with the concept of the questionnaire and who did not know the original document. This back translation was compared to the original version by a native English Speaker to ensure conceptual and semantic equivalence between the two versions. This pre-final version was evaluated by 25 patients for readability. This process is summarized in Fig. 1.

### Participants, setting and procedure

A total of 330 volunteers with Chronic Musculoskeletal Pain Disorders (CMPDs) were recruited consecutively from the community-based Physiotherapy Program at the Malaga University. Demographic variables of the subject sample can be found in Table 1. Subjects suffering from back, joint pain, and musculoskeletal diseases were included. Medical diagnoses were made by a physician at once of two primary care centers in Torremolinos, Malaga, Spanish National Health Service. Patients were excluded if they were aged < 18 years old, had poor Spanish language comprehension as required for the completion of the questionnaire, or suffered from any cognitive impairment. All subjects signed an informed consent to participate. All eligible participants filled out the FACS-Sp and the Spanish version of the Central Sensitization Inventory (CSI-Sp) [26].

**Table 1:** Descriptive anthropometric variables, painful area and FACS severity levels of the sample

	Mean ± SD
Age (Years)	55.04 ± 12.70
Height (m)	1.67 ± 0.09
Weight (Kg)	71.9 ± 14
BMI (Kg/m2)	25.61 ± 4.16
	**Percentage**
Gender	
Men	54.80
Women	45.20
Painful Areas	
Low back Pain	55.20
Neck Pain	34.30
Dorsal Back pain	11.50
Knee Pain	6.60
General Body: Arthrosis	5.40
Shoulder Pain	4.60
FACS severity levels	
Subclinical	26.90
Mild	49.90
Moderate	17.10
Severe	5.40
Extreme	0.80

### Patient-reported outcome measures

The FACS contains 20 separate items that are scored from 0 (“completely disagree”) to 5 (“completely agree”), with a total possible score of 100. Five severity levels are available for clinical interpretation: subclinical (0–20), mild (21–40), moderate (41–60), severe (61–80), and extreme (81–100) [16].

The CSI-Sp assesses 25 health-related symptoms common to Central Sensitization Syndromes (CSS). It contains 25 items that are scored from 0 (“never”) to 4 (“always”), with a total possible score of 100. The CSI-Sp has high internal consistency (α = 0.872) and test-retest reliability (r = 0.91) [27].

### Statistical methods

Normality of the distribution of data was determined by one-sample Kolmogorov-Smirnov test (significance < 0.05) for descriptive variables. Means and standard deviations of anthropometric variables were extracted for descriptive analyses.

### Structural validity

Factor structure was calculated from the Maximum Likelihood Extraction (MLE) by varimax rotation. Requirements for factor extraction as proposed in the literature [28], included Eigen value > 1.0 and accounting for > 10% of variance. A cutoff point of 0.32 item loading was considered the minimum load per item, according to Tabachnick and Fidell [29]. A minimum ratio of five participants-per-item was required, as detailed in the literature [28].

### Convergent validity

Convergent validity was calculated with Pearson correlation coefficients by P values (r,p) with the CSI-Sp. Along with FA, Central Sensitization is often present in individuals with CMPDs [30–32]. Therefore, the hypothesis was that the FACS-Sp would be positively correlated with CSI-Sp.

### Reliability

Internal consistency was obtained by Cronbach’s α coefficients at an anticipated value range of 0.80–0.95 [33, 34] for each factor, and ranges were expressed by Intraclass Correlation Coefficient (ICC 95%).

All statistical analyses were conducted using the Statistical Package for Social Science version 21.0 (SPSS 21.0) for Windows. Ethical clearance was approved by the Tribunal of Review of Human Subjects at the University of Malaga.

## Results

### Cross-cultural translation

The final Spanish version of FACS is shown in Additional file 1. Though most of the FACS-Sp items were translated without language difficulties or other conceptual misunderstanding, some items led to difficulties in translation. For example, the term “bad” employed in item 6, could be traduced like “malo” in Spanish, which implies and negative connotation. Therefore, the term “intense” fitted better than “strong” when describing pain. This translation was used to facilitate understating for patients. Difficulties were solved easily by consensus between the translators. The pre-final version was tested in 25 patients without any difficulty, so the pre-final version format was finally kept as FACS-Sp final version, and sample recruitment continued.

### Score distribution

No missing responses were found in the data collection. FACS-Sp scores ranged from 0 to 88 points, with a mean of 30.49 (±17.18). FACS severity level subgroups are displayed in Table 1.

### Structural validity

It was determined that the correlation matrix was adequate for the Maximum Likelihood Extraction from the results observed in Kaiser–Meyer–Oklin values (0.900) and the Bartlett’s test of sphericity (Chi-squared value =3259.568 and df 190, *p* < 0.001). Maximum Likelihood Extraction detected four components with Eigenvalues above 1, explaining 39.68, 11.57, 7.25, and 5.12% of the variance, respectively. The last two factors, however, accounted for less than 10% of the total variance, so they did not completed requirements for factor extraction, according to established methodology. Hence, a two factor solution was extracted according to the established criteria, explaining 48.75% of total variance. Table 2 shows each item loading on both extracted factors. It can be observed as load index values in factor 1 ranged from 0.081 in item 17 to 0.816 in item 8. In factor 2, values ranged from 0.848 in item 18 to 0.064 in item 1. Items 10, 14 and 20 cross-loaded on both factors. The Goodness-of-fit test revealed a Chi square of 685.027 (*p* < 0.000) after analysis.

**Table 2:** Variance explained, internal consistency and factor loading for item in both factor after maximum likehood extraction (FACS-Sp) and its original version

		FACS-Sp		FACS (original version)	
		Factor 1	Factor 2	Factor 1	Factor 2
Variance explained		39.68%	11.57%	39.7%	11.6%
Internal consistency		α = 0.904	α = 0.880	α = 0.92	
Item number					
1	Trato de evitar actividades y movimientos que empeoren mi dolor	**0.689**	0.064	**0.300**	0.115
2	Me preocupo por mi dolor	**0.699**	0.104	**0.674**	–0.011
3	Yo creo que mi dolor va a seguir empeorando hasta el punto de no poder hacer absolutamente nada.	**0.539**	0.263	**0.793**	–0.119
4	Me siento abrumado y con miedo cuando pienso en mi dolor	**0.630**	0.304	**0.786**	–0.175
5	Hay ciertas actividades que no intento por miedo de lastimarme o de volver a lastimarme	**0.747**	0.149	**0.755**	–0.016
6	Cuando mi dolor es realmente intenso, tengo otros síntomas como nausea, dificultad para respirar, el corazón late con fuerza, temblor y mareo	**0.350**	0.234	**0.294**	0.286
7	Es injusto que yo tenga que vivir con mi dolor	**0.614**	0.170	**0.628**	0.057
8	Hay ciertas actividades y movimientos que evito por miedo a que aumente mi dolor	**0.816**	0.121	**0.735**	–0.137
9	Debido a mi dolor, mi vida nunca será la misma	**0.661**	0.325	**0.713**	0.004
10	No tengo ningún control sobre mi dolor	0.468	0.309	**0.507**	0.279
11	Mi dolor me pone en riesgo de daños en el futuro (o volverme a dañar) por el resto de mi vida	**0.681**	0.268	**0.661**	0.250
12	Mi dolor es culpa de alguien más	0.195	0.336	**0.488**	0.103
13	El dolor que siento es una señal de advertencia que algo muy malo me está pasando	**0.426**	0.164	**0.552**	0.112
14	Nadie entiende lo grave que es mi dolor	0.402	0.383	**0.640**	0.155
15	…actividades intensas (como trabajo pesado de jardinería o mover muebles pesados) En la última semana, debido a mi dolor, he evitado las siguientes actividades...	**0.534**	0.157	–0.059	**0.815**
16	...actividades moderadas (como cocinar o limpiar el hogar)	0.228	**0.736**	–0.020	**0.795**
17	...actividades ligeras (como ir al cine o salir a comer)	0.081	**0.781**	–0.52	**0.667**
18	...todas mis tareas en el hogar y/o en el trabajo	0.127	**0.848**	0.149	**0.637**
19	...diversión y/o ejercicio (cosas que hago por diversión y por mantener mi buena salud)	0.194	**0.649**	0.199	**0.514**
20	...actividades donde tengo que usar mis parte(s) del cuerpo dañada	0.438	0.390	–0.080	**0.735**

### Convergent validity

The Pearson correlation for the FACS and CSI questionnaires was r = 0.414 (*p* < 0.001) in the total sample of 315 participants.

### Reliability

The FACS-Sp showed a high degree of internal consistency, as illustrated by the high.

Cronbach value α = 0.902 (ICC = 0.850–0.95) in factor #1 and α = 0.808 (ICC = 0.775–0.837) in factor #2.

## Discussion

The present study completed a cultural adaptation and validation of the FACS questionnaire to Spanish, resulting in a FACS-Sp version. In a first step, the translation process followed established guidelines explained in the literature, following a recommended direct- and reverse-translation- and back-translation methodology [25]. This process ensured the conceptual equivalence between terms employed in the original English version and the final version of FACS-Sp. In a second step, psychometric properties were evaluated in accordance with Costello and Osborne [28].

### Structural validity

The two-factor solution that emerged in the factor analysis accounted for 48.75% of the total variance. The English [35] and Serbian [36] versions of FACS also found a two-factor solution, which provides support for the construct validity of the FACS-Sp. In the English version, the two factors accounted for a very similar percentage of the total variance (51.54%). Also, like the FACS-Sp, the English version factor analysis detected four initial factors with Eigenvalues above 1 [35]. The FACS was designed to include four primary constructs: cognitive (pain catastrophizing), affective (pain-related fear/anxiety), and behavioral (avoidance), as well as the reason of avoidance (pain without fear; fear of pain; or fear of injury or re-injury) [16]. The presence of these constructs could explain the presence of four factor having eigenvalues > 1.0 in both the Spanish and English versions. However, only two factors achieved an explanation of the variance more than 10% in both versions, so only two were retained, as has been recommended. It was determined that factor 1 represented general FA and factor 2 represented the types of activities that one is avoiding.

Other FA-related questionnaires have shown several factors. FABQ contains two dimensions: While FA beliefs about work explain a 43.7% of the total variance, FA beliefs related to physical activity explain 16.5% [14]. The Spanish version of FABQ maintained 2 dimensions [20], while the German version split work-related dimension in two: work as cause of pain, explaining a 43.4% of the variance, and patients’ assumptions of their probable return to work (11.8% of the variance). Physical activity dimension explained 8.9% of the variance [37]. In the case of PCS, the original version was composed of 3 dimensions: rumination, magnification, and helplessness, which account respectively for 41, 10 and 8% of the total variance [13], with similar percentages in the Spanish version (39, 11 and 10%) [19]. The original TSK questionnaire contains two dimensions, named *Activity Avoidance* and *Harm,* as maintained in a 11-items Spanish version [18]. However, other versions present different factor solutions: 5 factors in a fibromyalgia syndrome population [38], 4 factors in its Dutch version by Principal Component Analysis [39], and 2 different factors (*Activity Avoidance* and *Pathological Somatic Focus*) in 17-items English version [40], which provides inconsistent findings.

Regarding items loading from the present version items related to cognitive and affective pain tended to load higher on factor 1, while items related to reasons of avoidance loaded higher on factor 2. In the English version of FACS, factor 1 was composed of items 1–14, and factor 2 was composed by items 15–20; while item 6 was cross-loaded in both factors but finally included in factor 1 [35]. In the Spanish version, factor 1 was composed of items 1–15, and factor 2 was composed of items 16–20 (see Table 2); while items 20, 14 and 10 were cross-loaded. Although item 6 showed a low load (0.350), the minimum of 0.32 [29] was achieved for factor 1. Therefore, it was not eliminated, keeping the same items as its original English version [35].

### Convergent validity

As expected, the FACS was positively correlated with the CSI, although this correlation was not strong (r = 0.414). This finding was expected as both questionnaires measure different construct. While the FACS measure FA, the CSI measures symptoms present in Central Sensitization Syndromes. However, the CSI was likely to converge (positive correlation) because both symptom dimensions often appear in CMPDs [30–32] and other chronic pain conditions [41, 42].

The convergent validity of CSI has previously been studied with questionnaires measuring FA, such as the PCS, showing similar results (*r* = .464; *p* < 0.001) in patients with chronic spinal pain [43]. Authors explained this association by the common variable of ‘Emotional distress’ which is assessed in both measures [44]. In patients with chronic nonspecific low back pain (LBP), CSI correlation with the PCS was higher (*r* = .518; *p* < 0.001), but lower and not significant with the TSK questionnaire (*r* = .348; *p* = 0.034) [45]. However, Pearson correlation was positive in both questionnaires, and patients with higher scores in pain catastrophizing and kinesiophobia showed higher degree of symptoms of CS measured by CSI. In addition to results from previous studies, It should be noted that FA is commonly identifies in patients with CSS [46], which would explain the correlation found between FACS and CSI.

### Internal consistency

The FACS-Sp showed a high internal consistency illustrated by α = 0.902 for factor 1 and 0.808 for factor #2. Similar results were found in the Serbian version, with higher internal consistency in factor 1 (α =0.904) than 2 (α = 0.880) The original version of FACS showed similar results for the entire questionnaire (α = 0.92), but separate results for each of the two factors were not reported [16].. This high internal consistency is similar to the original version of PASS (α = 0.94) [12]. Lower values were found for the FABQ and PCS [13, 14]. Other Spanish versions of pain-related FA questionnaires have also shown similar values, like the FABQ (α = 0.9337) [20]. Others have shown lower internal consistency, like the Spanish PCS (α = 0.79) [19] and Spanish TSK (α = 0.92 for chronic, and 0.781 for acute populations) [18].

### Clinical implications in primary care

According to the biopsychosocial model of chronic pain, psychological factors, like FA, contribute to the onset and progression of both pain and disability [47] and play a significant role in the transition between acute and chronic pain [48]. The sample from this study was suffering from CMPD, which represents a high cost in health care [49]. It should be noted that the mean FACS score in our study population was 30.49 ± 17.18, which corresponds to a mild severity level, according to recommended guideline from the original English version [16]. In the English version, two groups of patients with CMPD showed mean values of 67.90 ± 19.4 [16] and 68.2 ± 18.9 [35]. In the Serbian sample of subjects with CMPD, the mean value was 55.28 ± 22.53 [36]. Perhaps the Spanish subjects, with their lower FACS scores, were less chronic and/or less disabled than the English and Serbian populations. Or, perhaps cultural differences in self-report behaviour may have influenced the mean scores in these 3 subject populations.

Although avoidance behaviors may be adaptive in the context of acute pain, long-term avoidance can impair daily functioning and lead to physical disability [50]. As it is know that FA is associated with disability, FA is likely an important treatment target for reducing pain-related disability [50]. However, the translation of the guiding principles relating psychological factors to the clinic field have remained a challenge [47]. In this line, in 2011 the Institute of Medicine published a report on pain care, highlighting that a biopsychosocially-oriented approach, promoting patients’ self-management skills, was required for effectiveness of chronic pain treatment [51]. This paradigm promotes a multi-modal strategy including non-pharmacological pain treatment modalities [52]. Early intervention strategies, including screening and appropriate referrals, may help prevent patients with chronic illnesses from becoming disabled [53].

Approximately 55% of our sample was suffering from LBP. Evidence suggests that fear avoidance beliefs are prognostic for poor outcomes in subacute LBP, so early treatment, including interventions to reduce fear avoidance beliefs, may avoid delayed recovery and chronicity [54]. A recent systematic review concluded that targeting psychosocial predictors through clinical guidelines and a national strategy are need to support a cultural change in pain care [55]. Due to the new paradigm of pain, a biomechanical analysis is not comprehensive enough to identify subgroups [47] and credibility of subgroup claims in LBP trials is slow [56]. Therefore, LBP assessment should include the emotional and behavioral consequences of pain, which can contribute to the treatment outcomes [47]. Following this line, in primary care there are clinical prediction rules to identify patient prognosis to certain type of treatment [57]. Also, subgroup classification of patient with chronic LBP are available, based on a multifactorial approach, including psychological factors such as anxiety, depression, functional disability, pain, and FA beliefs [58]. Even clinical prediction models to inform clinical decision-making after some type of surgery are being developed, which includes FA [59]. Hence, FA assessment is crucial to guide treatment in primary care. Results from the present study offer a new screening tool for Spanish-speaking health providers in Spain and throughout Europe who wish to evaluate pain-related FA. It has demonstrated good psychometric properties, offers easy-to-interpret severity levels, and incorporates the important components of the most current FA model of Vlaeyen [6, 16].

### Study strengths

One of the strengths of the present study was the good psychometric results of the FACS-Sp, supporting previous research on the original English [35] and Serbian [36] versions. Our results suggest that reliable score comparisons can be made between different language-speaking populations in different areas of the world and that the FACS-Sp is appropriate for Spanish-speaking populations in Europe.

### Limitations

As a limitation, there was a lack of longitudinal data, so it did not include test-retest reliability, responsiveness and error scores or provide information on minimal clinically important difference values.

## Conclusion

This study reports for the first time the psychometric properties of the Spanish version of the FACS (FACS-Sp). The reliability, in terms of internal consistency, and structural validity were comparable to the original English version [16], ensuring conceptual equivalence. The FACS-Sp provides a reliable and potentially useful FA measures for European-style Spanish-speaking populations. The fact that Spanish is the second largest geographical language [60] makes this new FACS-Sp especially important for more wide-spread international use. Finally, future research on the FACS-Sp should evaluate test-re-test reliability, treatment responsiveness and psychometric comparisons with other translated versions. In primary care, FA assessment is crucial in clinical decision-making and treatment planning. The FACS-Sp allows a quick and easy patient-reported measure of FA in Spanish speaker populations.

## Supplementary information

**Additional file 1.**

## Data Availability

The datasets analyzed during the current study are available from the corresponding author on reasonable request.

## Abbreviations

BMI: Body Mass Index
CSI-Sp: Spanish version of the Central Sensitization Inventory
CSS: Central Sensitization Syndromes
FA: Pain-related Fear Avoidance
FABQ: Fear-Avoidance Beliefs Questionnaire
FACS: Fear-Avoidance Components Scale (FACS)
FACS-Sp: Fear-Avoidance Components Scale European-style Spanish
MLE: Maximum Likelihood Extraction
PASS: Pain Anxiety Symptoms Scale
PCS: Pain Catastrophizing Scale
PROMS: Patient-Reported Outcome Measures
TSK: Tampa Scale for Kinesiophobia
