# Supplementary material for: Cross-cultural adaptation and validity of the Spanish fear-avoidance components scale and clinical implications in primary care
Source: BMC Fam Pract. 2020 Feb 27;21:44. doi: 10.1186/s12875-020-01116-x (PMC7047382; doi:10.1186/s12875-020-01116-x)
Supplement: Supplementary file 1 — Additional file 1. [file 12875_2020_1116_MOESM1_ESM.docx]

ESCALAS DE COMPONENTES DE EVITACIÓN DEL MIEDO AL DOLOR

Nombre:__________________________________________ Fecha:_______________________

Por favor rodee la respuesta correcta para cada uno de los enunciados.

INSTRUCCIONES: La gente responde al dolor de diferentes maneras. Queremos saber qué piensa y siente sobre su dolor y cómo afecta su nivel de actividad. Por favor, piensa sobre cómo ha estado en la última semana y conteste el cuestionario rodeando del 0 al 5 de acuerdo a la escala que se muestra a continuación.

| En la última semana, ¿Cuánto está de acuerdo con las siguientes afirmaciones? | | Completamente de acuerdo | En su mayor parte de acuerdo | Ligeramente de acuerdo | Ligeramente de desacuerdo | En su mayor parte desacuerdo | Completamente desacuerdo |
| --- | --- | --- | --- | --- | --- | --- | --- |
| 1 | Trato de evitar actividades y movimientos que empeoren mi dolor | 5 | 4 | 3 | 2 | 1 | 0 |
| 2 | Me preocupo por mi dolor | 5 | 4 | 3 | 2 | 1 | 0 |
| 3 | Yo creo que mi dolor va a seguir empeorando hasta el punto de no poder hacer absolutamente nada | 5 | 4 | 3 | 2 | 1 | 0 |
| 4 | Me siento abrumado y con miedo cuando pienso en mi dolor | 5 | 4 | 3 | 2 | 1 | 0 |
| 5 | Hay ciertas actividades que no intento por miedo de lastimarme o de volver a lastimarme | 5 | 4 | 3 | 2 | 1 | 0 |
| 6 | Cuando mi dolor es realmente intenso, tengo otros síntomas como nausea, dificultad para respirar, el corazón late con fuerza, temblor y mareo | 5 | 4 | 3 | 2 | 1 | 0 |
| 7 | Es injusto que yo tenga que vivir con mi dolor | 5 | 4 | 3 | 2 | 1 | 0 |
| 8 | Hay ciertas actividades y movimientos que evito por miedo a que aumente mi dolor | 5 | 4 | 3 | 2 | 1 | 0 |

Continuar….

| En la última semana, ¿Cuánto está de acuerdo con los siguientes enunciados? | | Completamente de acuerdo | En su mayor parte de acuerdo | Ligeramente de acuerdo | Ligeramente de desacuerdo | En su mayor parte desacuerdo | Completamente desacuerdo |
| --- | --- | --- | --- | --- | --- | --- | --- |
| 9 | Debido a mi dolor, mi vida no es la misma | 5 | 4 | 3 | 2 | 1 | 0 |
| 10 | No tengo ningún control sobre mi dolor | 5 | 4 | 3 | 2 | 1 | 0 |
| 11 | Mi dolor me pone en riesgo de daños en el futuro (o volverme a dañar) por el resto de mi vida | 5 | 4 | 3 | 2 | 1 | 0 |
| 12 | Mi dolor es culpa de alguien más | 5 | 4 | 3 | 2 | 1 | 0 |
| 13 | El dolor que siento es una señal de advertencia que algo muy malo me está pasando | 5 | 4 | 3 | 2 | 1 | 0 |
| 14 | Nadie entiende lo grave que es mi dolor | 5 | 4 | 3 | 2 | 1 | 0 |

| Termine cada una de las siguientes frases, empezando con el siguiente enunciado:  En la última semana, debido a mi dolor, he evitado las siguientes actividades: | | Completamente de acuerdo | En su mayor parte de acuerdo | Ligeramente de acuerdo | Ligeramente de desacuerdo | En su mayor parte desacuerdo | Completamente desacuerdo |
| --- | --- | --- | --- | --- | --- | --- | --- |
| 15 | … actividades intensas (como trabajo pesado de jardinería o mover muebles pesados) | 5 | 4 | 3 | 2 | 1 | 0 |
| 16 | … actividades moderadas (como cocinar o limpiar el hogar) | 5 | 4 | 3 | 2 | 1 | 0 |
| 17 | … actividades ligeras (como ir al cine o salir a comer) | 5 | 4 | 3 | 2 | 1 | 0 |
| 18 | … todas mis tareas en el hogar y/o en el trabajo | 5 | 4 | 3 | 2 | 1 | 0 |
| 19 | … diversión y/o ejercicio (cosas que hago por diversión y por mantener mi buena salud) | 5 | 4 | 3 | 2 | 1 | 0 |
| 20 | … actividades donde tengo que usar mi/s parte/s del cuerpo dañada/s | 5 | 4 | 3 | 2 | 1 | 0 |
|  | Resultado total: __________________________________________________________ | | | | | | |
